# Selection of Multidrug-Resistant Bacteria in Medicated Animal Feeds

**DOI:** 10.3389/fmicb.2019.00456

**Published:** 2019-03-06

**Authors:** Emily E. F. Brown, Ashley Cooper, Catherine Carrillo, Burton Blais

**Affiliations:** Research and Development Section, Ottawa Laboratory Carling, Science Branch, Canadian Food Inspection Agency, Ottawa, ON, Canada

**Keywords:** antibiotic, sulfonamide, carbapenem, resistance, selection, cross-resistance, medicated feeds

## Abstract

Exposure to antimicrobial resistant (AMR) bacteria is a major public health issue which may, in part, have roots in food production practices that are conducive to the selection of AMR bacteria ultimately impacting the human microbiome through food consumption. Of particular concern is the prophylactic use of antibiotics in animal husbandry, such as the medication of feeds with sulfonamides and other antibiotics not considered clinically relevant, but which may nonetheless co-select for multi-drug resistant (MDR) bacteria harboring resistance to medically important antibiotics. Using a MDR *Klebsiella pneumoniae* strain exhibiting resistance to sulfonamides and beta-lactams (including carbapenem) as a model, we examined the ability of non-medicated and commercially medicated (sulfonamide) animal feeds to select for the model strain when inoculated at low levels by measuring its recovery along with key AMR markers, *sul1*(sulfonamide) and *blaKPC-*3 (meropenem), under different incubation conditions. When non-medicated feeds were supplemented with defined amounts of sulfadiazine the model strain was significantly enriched after incubation in Mueller Hinton Broth at 37°C overnight, or in same at room temperature for a week, with consistent detection of both the *sul1* and *blaKPC-*3 markers as determined by polymerase chain reaction (PCR) techniques to screen colony isolates recovered on plating media. Significant recoveries of the inoculated strain and the *sul1* and *blaKPC-*3 markers were observed with one of three commercially medicated (sulfamethazine) feeds tested under various incubation conditions. These results demonstrate that under certain conditions the prophylactic use of so-called non-priority antibiotics in feeds can potentially lead to co-selection of environmental AMR bacteria with resistance to medically important antibiotics, which may have far-reaching implications for human health.

## Introduction

The occurrence of food-borne bacteria with anti-microbial resistance characteristics (e.g., resistance to therapeutic antibiotics) is widely regarded as a serious public health threat as foods constitute a primary niche for human exposure to environmental microbiota. Outbreaks of foodborne illness caused by antimicrobial resistant (AMR) bacteria are becoming more common, for example, multi-drug resistant (MDR) strains of *Klebsiella pneumoniae* producing extended-spectrum β-lactamases were implicated in foodborne outbreaks in Spain (Calbo et al., 2011), and a recent outbreak of salmonellosis in the US was linked to chicken contaminated with an MDR strain of *Salmonella* Heidelberg (Gieraltowski et al., 2016). The question of how pathogenic bacteria acquire antibiotic resistance is the subject of intense research, and one possibility is that commensal bacteria occurring in food production environments may serve as a reservoir for AMR genes residing on mobile genetic elements which can be passed on to human pathogens (Economou and Gousia, 2015).

In North America it has been common practice to supplement livestock feeds with antibiotics for growth-promotion and prophylaxis (disease prevention). There is some evidence indicating that this practice may select for the acquisition of AMR traits and their transfer to the microbiota of food production animals such as cattle, chickens and pigs (Economou and Gousia, 2015; Jahanbakhsh et al., 2015; Agga et al., 2016), and to human pathogenic bacteria (Marshall and Levy, 2011; Ter Kuile et al., 2016). To minimize the risk of fostering the development of AMR to medically important antibiotics the tendency has been to use those considered lower priority for clinical applications, for example, sulfonamides and tetracyclines. As a precautionary measure in 2006 the European Union banned the labeling of antibiotics as growth promotants in feeds (European Union, 2005; Levy, 2014), and Canada followed suit in 2017 (Mehrotra and Ireland, 2017). However, there remains considerable scope for the use of antibiotics in feeds as prophylactic agents to treat or prevent infections in food production animals (FDA, 2013). One potential pitfall of using non-priority antibiotics prophylactically is the fact that some environmental bacteria exhibit MDR traits due to carriage of multiple resistance genes on plasmids or within a combination of plasmids and chromosomal loci (Chakraborty, 2017). For MDR bacteria application of selective pressure for one trait may result in co-selection of other resistance genes harbored in a single cell. In the present context, co-selection occurs when an MDR bacterial population is expanded on exposure to a single antibiotic for which it carries a resistance gene, resulting in a concomitant increase of any other AMR genes harbored within the same cells.

We were interested in studying co-selection of a medically important resistance trait in an environmental MDR bacterial species capable of entering the food production chain (e.g., via animal feeds), under selection conditions involving the presence of a typical antibiotic used in commercially medicated feeds. For this purpose, we designated an MDR *K. pneumoniae* isolate recovered from raw sewage influent as a model for a naturally occurring environmental bacterium with the potential to serve as a reservoir for the transfer of AMR genes to the food animal microbiome. *K. pneumoniae* is considered an important source of AMR genes that can be readily transferred to other bacteria, including commensals and pathogens found in food production environments (Berendonk et al., 2015). The particular model strain chosen was determined to possess genes specifying resistance to sulfonamides, commonly used in feeds, as well as medically important beta-lactams, including meropenem, which is a member of the high priority carbapenem antibiotic class. A key objective of this study was to determine if this strain could be enriched after incubation under different conditions in animal feeds containing sulfadiazine, and also, whether the enriched bacteria carried both sulfonamide and meropenem resistance genes.

## Materials and Methods

### Bacterial Strains

Bacterial strains were routinely grown on MacConkey (MAC) agar (BD Difco Inc., Belgium) at 37°C for 16–18 h. Suspensions for inoculation were prepared by growth in 10 mL Mueller Hinton Broth (MHB) (Oxoid Ltd., United States) at 37°C for 16–20 h, and counted by plating serial dilutions on nutrient agar (NA) (Oxoid Ltd., Basingstoke, United Kingdom) plates which were incubated for 16–20 h at 37°C. Bacterial isolates recovered from feed samples were identified using a VTEK 2 GN ID Card (bioMérieux, Canada) following the manufacturer’s instructions.

Two *K. pneumoniae* strains were used for feed inoculation experiments. One strain (OLC-1237) was from the culture collection of the Ottawa Laboratory Carling (Canadian Food Inspection Agency) and determined to be susceptible to sulfonamides on the basis of phenotypic and genomic analyses (see below); whereas, another strain, determined to be MDR (including resistance to sulfonamides and carbapenem) on the basis of genomic and phenotypic analyses (Table 1), was isolated from an Ottawa, Ontario, waste water treatment plant using the method described by Zurfluh et al. (2013), and designated OLC-2685. Briefly, 250 ml of influent sample was concentrated by filtration through 0.80 and 0.22 μm S-Pak membrane filters (Millipore, France). Filters were aseptically transferred to a sterile stomacher bag containing 25 ml modified tryptone soya broth (mTSB) (Oxoid). Filters were rinsed by manually massaging stomacher bags for 5 min to release collected bacterial cells. An aliquot from each rinse was serially diluted in sterile phosphate buffered saline (PBS), and dilutions were plated in triplicate on MacConkey agar (Sigma-Aldrich, St. Louis, MO, United States) containing 4 μg/ml meropenem (MACMER) (USP, United States) to select for carbapenem resistant Gram-negative bacteria, and incubated at 37°C for 16–20 h. The *K. pneumoniae* isolate selected was identified by whole genome sequence analysis and subjected to AMR gene characterization (below). Antimicrobial susceptibility testing of the strains was determined using the Sensititre CMV4AGNF susceptibility plate (Thermo Fisher Scientific, United States) following the manufacturer’s instructions. Antimicrobial susceptibility testing of bacterial strains was also performed using the Sensititre CMV4AGNF susceptibility plate (Thermo Fisher Scientific, United States), as well as VITEK 2 AST-GN98 and –N208 cards (bioMérieux), following the manufacturers’ instructions.

**Table 1:** ResFinder analyses of *K. pneumoniae* strains used in this study.

Antimicrobial	OLC-1237	OLC-2685
β-Lactams	*BlaSHV-11*-like	*blaCARB-2, blaKPC-3, blaOXA-9, blaSHV-11, blaTEM-1A*-like
Aminoglycosides		*aac(6’)-Ib-like, aadA1, aadA2, aph(3’)-Ia, strA*-like, *strB*-like
Sulfonamides		*sul1*
Quinolones	*OqxA-like, oqxB*	*oqxA, oqxB*
Fosfomycin	*fosA-*like	*fosA*-like
Macrolides		*mph(A), mph(E), msr(E)*
Trimethoprim		*dfrA18*-like
Phenicols		*cmlA1*-like
Fluoroquinolones		*aac(6’)Ib-cr*-like


### Whole Genome Sequence Analysis

Whole genome sequence analysis of *K. pneumoniae* strains OLC-1237 and OLC-2685 was performed to determine the presence of AMR genes and confirm the bacterial species identity. Genomic DNA was extracted from bacteria grown in MHB (Oxoid) at 37°C for 16–20 h using the Promega Maxwell 16 Cell DNA purification kit (Promega, United States). Sequencing libraries were constructed using the Nextera XT DNA sample preparation kit (Illumina, Inc., United States) and paired-end sequencing was performed on the Illumina MiSeq platform using 600 cycle MiSeq reagent kits (v3) with 6% PhiX control. Quality of raw sequencing reads was assessed with FastQC version 0.11.4 (Andrews, 2010), quality trimmed with bbduk version 37.66 (Bushnell, 2016), and error corrected using BayesHammer (Nikolenko et al., 2013) included with the SPAdes genome assembler version 3.7.1 (Nurk et al., 2013). Contigs were assembled from the trimmed and error-corrected reads using SPAdes version 3.7.1 (Nurk et al., 2013) with the “careful” setting enabled. Assembly quality was assessed with qualimap version 2.2 (Okonechnikov et al., 2015), and quast version 2.3 (Gurevich et al., 2013). MASH version 2.0 (Ondov et al., 2016), was used to determine the genera of the isolates. Core genomes, plasmid and prophage complement, vector contamination, in addition to 16S sequencing, MLST and rMLST profiles, as well as the presence of markers associated with virulence, and antimicrobial resistance were calculated using custom Python scripts^1^. AMR gene detection was additionally conducted using the Center for Genomic Epidemiology ResFinder webtool (v2.1^2^) with the default settings for gene identity threshold (90 %) and minimum length (60%) (Zankari et al., 2012). Plasmid maps were generated using PlasMapper (Dong et al., 2004). The raw sequence data for strains OLC-1237 and OLC-2685 can be downloaded from the Sequence Read Archive using accessions SRR7788649 and SRR7796513, respectively.

### Animal Feeds and Sulfonamide Residue Testing

A variety of feeds obtained through Canadian Food Inspection Agency testing programs and analyzed for sulfonamide content were used for this study. Feed OTT-FE-2017-0819 was a hog grower pellet with no detectable antibiotic content and determined to contain 10^5^ cfu/g total background microbiota. OTT-FE-2016-0651 was rolled corn containing 0.76 mg/kg sulfamethazine and determined to contain 9 × 10^4^ cfu/g total background microbiota. OTT-FE-2017-0006 and OTT-FE-2017-0007 were both pig starter meals containing 110 mg/kg sulfamethazine, and determined to contain 6 × 10^4^ and 9 × 10^4^ cfu/g total background microbiota, respectively. All feeds were provided in ground form. The presence of antibiotics and their concentrations were determined by liquid chromatography with post-column derivatization as previously described (Smallidge and Albert, 2000). Background microbiota counts were determined by plating serial dilutions of feed-in-broth suspension (below) on NA and incubating for 16–20 h at 37°C.

### Enrichment of *Klebsiella pneumoniae* in Non-medicated Feed Mixed With Defined Levels Sulfadiazine

Ten grams sub-samples of a non-medicated feed (OTT-FE-2016-0819) were combined with 90 mL MHB to which defined levels (0, 0.5, or 2 mg/mL) of sulfadiazine (Sigma-Aldrich, St. Louis, Mo, United States) were added. The feed suspensions were then inoculated with 0 or 149 cfu of OLC-1237 (sensitive strain) or 150 cfu of OLC-2685 (resistant strain) and mixed. Two sets of samples were prepared in this manner, and one was incubated at 35°C for 16–20 h, while the other was left in the dark at room temperature (22°C) for a period of 7 days. After incubation decimal dilutions of the suspensions were plated on regular MAC agar, and incubated at 37°C for 16–20 h, followed by assay of the colonies (minimum 30 colonies sampled per plate) using the PCR methods described below.

### Enrichment of MDR *K. pneumoniae* in Commercially Medicated Feeds

Feed samples commercially medicated with sulfadiazine (OTT-FE-2016-0651, OTT-FE-2017-0006 or OTT-FE-2017-0007) were combined at a ratio of 10 g with 90 mL of MHB as above. The feed suspension was then inoculated with 0 or 140 cfu of OLC2685. Two sets of samples were prepared in this manner, and one was incubated at 37°C for 16–20 h, while the other was left in the dark at room temperature (22°C) for a period of 7 days. After incubation decimal dilutions of the suspensions were plated on MAC agar, and incubated at 37°C for 16–20 h, followed by assay of the colonies (minimum 30 colonies sampled per plate) using the PCR methods described below.

### Enrichment of MDR *Klebsiella pneumoniae* in “Moist” Commercially Medicated Feed

One gram portions of a feed sample (OTT-FE-2016-0651) were placed in individual sterile test tubes then inoculated with 100 μl of phosphate-buffered saline (PBS) containing 0 or 113 cfu of OLC-2685. To create a moist environment in the feed samples, 1 mL of sterile PBS was added to each tube, which were then placed in a 37°C incubator with 58–62% humidity for 6 days. Samples were then resuspended in 5 mL of PBS and serial dilutions of each suspension were plated on MAC agar, and incubated at 37°C for 16–20 h, followed by assay of the colonies (minimum 30 colonies sampled per plate) using the PCR methods described below.

### Polymerase Chain Reaction (PCR) Colony Analyses

#### Sulfonamide and Carbapenem Resistance Gene Assays

Bacterial colonies recovered from feeds (below) were assayed by separate PCR techniques targeting the key genes conferring resistance to sulfonamides (*Sul1*) (Frank et al., 2007) and carbapenem (*blaKPC-3*) (Bogaerts et al., 2013; Table 2), which were determined by ResFinder analysis to occur in the MDR *K. pneumoniae* strain (OLC-2685) used in this study (Table 1).

**Table 2:** Primers used for PCR colony assays.

Target gene	Primer Sequence (5′ to 3′)^a^	Amplicon Size (bp)	Source
*blaKPC-3*	TCGCCGTCTAGTTCTGCTGTCTTG	353	Bogaerts et al., 2013
	ACAGCTCCGCCACCGTCAT		
*Sul1*	CGGCGTGGGCTACCTGAACG	433	Frank et al., 2007
	GCCGATCGCGTGAAGTTCCG		
OLC-1237	AGGACGCAGACCACCTATCT	314	This study
	TACTGACGCGCGCAGATATT		
OLC-2685	ACGTTTACGCTACCGGAGGA	299	This study
	ATCTCCCCGCCAAGTGAATG		


*^a^Degenerate bases: Y, pyrimidine; R, purine*.

Bacterial colony lysates were prepared by suspension of a colony in 200 μl of 1% (v/v) Triton X-100 (Sigma-Aldrich), followed by heating at 100°C for 10 min, and 2 μL of lysate was combined with of 25 μL of reaction mixture containing 2.5 units HotStar Taq, 1.6 X HotStar PCR buffer (giving a final MgCl_2_ concentration of 2.4 mM) (Qiagen Inc., Canada), 200 μM of each dNTP,and 0.2 μM of each (forward and reverse) primer (Table 2). The PCR was carried out in a Mastercycler gradient thermal cycler (Eppendorf, Germany) using the following conditions: an initial denaturation cycle at 94°C for 15 min, followed by 35 cycles of denaturation at 94°C for 30 s, primer annealing at 60°C for 30 s, and elongation at 72°C for 1 min 30 s, with an additional 2 min at 72°C following the last cycle. Amplicons were analyzed using the QIAxcel (Qiagen) system (following the manufacturer’s instructions).

#### Strain-Specific PCR Primers

The recovery of inoculum strains used in the above feed enrichment studies was confirmed by assaying colonies using separate strain-specific PCR methods targeting OLC1237 and OLC2685. Strain-specific DNA sequences were determined *in silico* using whole genome sequence data from target strains OLC1237 and OLC2685 analyzed by the SigSeekr tool previously described for the identification of strain-specific signature sequences for PCR primer development (Knowles et al., 2015).

Primers for the strain-specific PCR methods (Table 2) were produced from the signature sequences identified by SigSeekr using the NCBI primer-BLAST tool (Ye et al., 2012). The specificity of primers for the respective target strains was verified by ascertaining that the expected PCR products under optimized PCR conditions (above) were only obtained with the intended target strains, and not with 21 different non-target *K. pneumoniae* and 7 different *K. oxytoca* strains, as well as 12 different *Escherichia coli* and 4 *Hafnia alvei* strains from the OLC culture collection.

### Statistical Analyses

Data were analyzed using the two-proportions *z*-test with R version 3.3.1 (R Core Team, 2013). PCR positive results were considered successes out of *n* CFU tested for each experiment. One-tailed *z*-tests were used test whether the proportion of OLC-2685 positives was greater in higher concentrations of sulfadiazine (non-medicated feed experiments, Tables 3, 4) and to compare inoculated and uninoculated commercially medicated samples (Tables 5–7). A two sample two-tailed *z*-test was used to compare growth of OLC-2685 and OLC-1237 in the absence of sulfadiazine (non-medicated feed).

**Table 3:** Recovery of inoculated *K. pneumoniae* strains grown at 37°C overnight in a non-medicated feed.

Inoculum	Sulfadiazine (mg/mL)	PE cell density (cfu/mL)^a^	No. of colonies assayed^b^	PCR-positive colonies (%)^c^			
				OLC-2685	OLC-1237	*blaKPC-3*	*Sul1*
Uninoculated	0	10^8^	30	0	0	0	0
	0.5	10^7^	30	0	0	0	100
	2	10^4^	33	0	0	0	0
OLC-2685	0	10^8^	30	0	0	0	0
	0.5	10^7^	30	10	0	10	70
	2	10^5^	34	100	0	100	100
OLC-1237	0	10^8^	30	0	13	0	0
	0.5	10^7^	30	0	0	0	0
	2	10^4^	33	0	0	0	0


*^a^PE, post-enrichment (total density of bacteria obtained after enrichment). ^b^Number of colonies isolated on plating media tested by PCR. ^c^Percentage of colonies giving PCR-positive results*.

**Table 4:** Recovery of inoculated *K. pneumoniae* strains grown at room temperature for a week in a non-medicated feed.

Inoculum	Sulfadiazine (mg/mL)	PE cell density (cfu/mL)^a^	No. of colonies assayed^b^	PCR-positive colonies (%)^c^			
				OLC-2685	OLC-1237	*blaKPC-3*	*Sul1*
Uninoculated	0	10^7^	30	0	0	0	0
	0.5	10^6^	30	0	0	0	17
	2	10^4^	33	0	0	0	30
OLC-2685	0	10^7^	30	3	0	3	3
	0.5	10^6^	30	20	0	20	33
	2	10^4^	34	79	0	79	91
OLC-1237	0	10^7^	30	0	7	0	0
	0.5	10^6^	30	0	0	0	53
	2	10^4^	34	0	0	0	23


*^a^PE, post-enrichment (total density of bacteria obtained after enrichment). ^*b*^Number of colonies isolated on plating media tested by PCR. ^*c*^Percentage of colonies giving PCR-positive results*.

**Table 5:** Recovery of inoculated MDR *K. pneumoniae* strain grown at 37^o^C in commercially medicated feeds.

Feed	Inoculum	PE cell density (cfu/mL)^a^	No. of colonies assayed^b^	PCR-positive colonies (%)^c^		
				OLC-2685	*blaKPC-3*	*Sul1*
OTT-FE-2016-0651	None	10^5^	30	0	0	0
	OLC-2685	10^7^	34	100	100	100
OTT-FE-2017-0006	None	10^8^	30	0	0	0
	OLC-2685	10^8^	30	7	7	7
OTT-FE-2017-0007	None	10^8^	30	0	0	0
	OLC-2685	10^8^	30	0	0	0


*^a^PE, post-enrichment (total density of bacteria obtained after enrichment). ^*b*^Number of colonies isolated on plating media tested by PCR. ^*c*^Percentage of colonies giving PCR-positive results*.

**Table 6:** Recovery of inoculated MDR *K. pneumoniae* strain grown at room temperature for a week in commercially medicated feeds.

Feed	Inoculum	PE cell density (cfu/mL)^a^	No. of colonies assayed^b^	PCR-positive colonies (%)^c^		
				OLC-2685	*blaKPC-3*	*Sul1*
OTT-FE-2016-0651	None	10^9^	30	0	0	0
	OLC-2685	10^9^	34	18	18	18
OTT-FE- 2017-0006	None	10^9^	30	0	0	0
	OLC-2685	10^9^	30	0	0	0
OTT-FE-2017-0007	None	10^9^	30	0	0	0
	OLC-2685	10^9^	34	0	0	0


*^a^PE, post-enrichment (total density of bacteria obtained after enrichment). ^*b*^Number of colonies isolated on plating media tested by PCR. ^*c*^Percentage of colonies giving PCR-positive results*.

**Table 7:** Recovery of inoculated MDR *K. pneumoniae* strain incubated at 37°C for a week in a moist commercially medicated feed.

Inoculum	PE cell density (cfu/mL)^a^	No. of colonies assayed^b^	PCR-positive colonies (%)^c^		
			OLC-2685	*blaKPC-3*	*Sul1*
None	10^7^	30	0	0	0
OLC-2685	10^7^	36	100	100	100


*^a^PE, post-enrichment (total density of bacteria obtained after enrichment). ^*b*^Number of colonies isolated on plating media tested by PCR. ^*c*^Percentage of colonies giving PCR-positive results*.

## Results and Discussion

### Model *K. pneumoniae* Strains

Both strains used in this study were determined to harbor a variety of genes conferring resistance to antibiotics (Table 1). Strain OLC-1237, which is a legacy strain from the CFIA culture collection, exhibited the presence of a small number of resistance genes specifying resistance to β-lactams, quinolones and fosfomycin, but did not contain any known sulfonamide resistance genes, nor did it exhibit phenotypic resistance to sulfadiazine, and for this reason was selected to serve as a (non-resistant) control in these studies. The MDR strain OLC-2685, recently isolated from a waste treatment plant, constitutes a “real world” strain which has not been laboratory-adapted, and therefore is highly representative of an environmental bacterium which can potentially occur in animal feeds. This strain was found to carry many different resistance genes, key among which (for present purposes) were genes specifying resistance to sulfonamides (*sul1*) and meropenem (*blaKPC-3*) (Table 1), the resistance phenotypes for which were confirmed (not shown). Genomic analysis of strain OLC-2685 determined the presence of multiple copies of *sul1* (though their location on the chromosome or plasmids could not be ascertained) and that the *blaPKC-3* gene was located on the *Klebsiella* plasmid pRYCKPC3.1, which did not contain a *sul1* gene, indicating that the two genes were not genetically linked. We were also able to determine the presence of genomic signature sequences for the development of strain-specific PCR methods (Table 2) as an aid in determining their selection and recovery in inoculated feed samples. PCR methods targeting the *blaKPC-3* and *Sul1* genes found in OLC-2685 (Table 2) were used as a means of verifying their selection or co-selection during the enrichment process.

### Enrichment in Non-medicated Feed

We were interested in studying the enrichment of model MDR and sulfonamide-sensitive *K. pneumoniae* strains in feeds having unselected background microbiota, and to which defined amounts of sulfadiazine could be added at the start of the selection process. For these experiments we used sulfadiazine as a typical representative of the sulfonamide family of antibiotics. The amounts of sulfadiazine used were selected to bracket the known minimum inhibitory concentration range for sulfonamide-resistant *K. pneumoniae* (typically >2 mg/mL) (Lin et al., 2016).

Portions of a non-medicated hog pellet feed sample (OTT-FE-2017-0819) were inoculated with a relatively low number of *K. pneumonia* cells (approximately 2–3 orders of magnitude lower than the level of background bacteria), either OLC-1237 (sulfadiazine-sensitive) or OLC-2685 (MDR, including sulfadiazine- and carbapenem-resistant), or left un-inoculated, then mixed with nutrient-rich MHB containing various levels of sulfadiazine. Samples incubated at 37°C to foster rapid growth exhibited no discernible enrichment of OLC-2685 in the absence of sulfadiazine when colonies isolated on plating media from the enrichment broth cultures were assayed by PCR for the strain-specific, *Sul1* and *blaKPC-3* resistance markers (Table 3). This may be due to overgrowth by background bacteria, as evidenced by the fact that the final enrichment broth cultures reached high cell densities matching those observed for the un-inoculated portion. On the other hand, when sulfadiazine was added to the enrichment broth at a low concentration (0.5 mg/mL), the inoculated strain OLC-1237 was not recovered, whereas it was possible to recover the inoculated OLC-2685 strain, albeit at the low rate of 10 % of all colonies isolated on plating media (*p* = 0.1181). In this instance, all of the colonies bearing the OLC-2685 genetic marker also bore the *Sul1* and *blaKPC-3* genes, as determined by PCR. The majority of the remainder of the colonies recovered on the plates were background bacteria bearing the *Sul1* gene, which presumably was present among the background bacteria initially and enabled their selection in the presence of sulfadiazine. Analysis of twelve of these isolates revealed their identity as *K. pneumoniae* and *Enterobacter cloacae*.

When the sample inoculated with OLC-2685 was grown in the presence of a high concentration (2 mg/mL) of sulfadiazine all of the bacteria recovered from the enrichment broth on plating media proved to be the inoculated strain, as evidenced by detection of the strain-specific marker and the two antibiotic resistance genes in all of the colonies (*p* < 0.0001, compared to both uninoculated samples and those inoculated with OLC-1237) (Table 3). The ability of the inoculated strain to outcompete the background bacteria, even those bearing the *Sul1* gene, may be attributable to the fact the OLC-2685 was found by genomic analysis to harbor multiple copies of this gene, which may have provided a survival advantage in the presence of a higher concentration of sulfadiazine.

The total cell densities achieved in the enrichment broth cultures were consistently lower (by one to four orders of magnitude) in the presence of sulfadiazine, indicating that the antibiotic effectively suppressed growth of the background bacteria. Those cells which did grow at the lower sulfadiazine concentration were found to harbor the *Sul1* gene (Table 3), demonstrating the enrichment effect of sulfadiazine when conditions were suitable for the growth of bacteria in the feed sample. No significant difference was observed for recovery of OLC-1237 and OLC-2685 in the absence of sulfadiazine in non-medicated feed (*p* = 0.1205 and 1 for 37°C and room temperature, respectively) (Tables 3, 4). The inability to recover strain OLC-1237 – which is of the same species as the MDR strain – in the presence of sulfadiazine (Table 3) further supports the notion that selective enrichment of the MDR *K. pneumoniae* strain was due to the presence of its sulfonamide resistance trait, and not necessarily because *K. pneumoniae* are more competitive. It should be noted that in every instance (even in the absence of an inoculated strain) colonies were recovered at all concentrations of sulfadiazine, and the lack of a detectable *Sul1* gene in these colonies suggests that they were either species with intrinsic resistance to sulfonamides or bearing alternative resistance factors.

The implications of these experimental results are two-fold: (1) the presence of sulfadiazine in a feed sample imposes selective pressure which favors the outgrowth of *K. pneumoniae* (and possibly other bacteria) possessing resistance associated with *Sul1*, even in the presence of high levels of background bacteria; and (2) selection on the basis of a single resistance trait causes co-selection of other traits present in the same cell, such as the gene specifying carbapenem resistance, even though the gene resides on a separate genetic element (plasmid).

Selection of one strain in a complex population may be influenced by growth dynamics of the broader population. Therefore, we examined the impact of slow growth by carrying out the enrichments under sub-optimal conditions, where the samples were left at room temperature for 7 days. Under these conditions, a similar pattern of selective enrichment of OLC-2685 was observed as with enrichment under fast growth conditions, with all colonies recovered in the presence of sulfadiazine bearing the strain-specific marker as well as the *Sul1* and *blaKPC-3* markers (Table 4), demonstrating the same selective enrichment and resistance gene co-selection phenomenon as before (Table 3). Altered population growth dynamics may account for quantitative differences in the results obtained with the two growth conditions, as well as the differential recoveries of background bacteria bearing *Sul1*.

### Enrichment in Commercially Medicated Feeds

The observation that the endogenous feed community contains bacteria with sulfonamide resistance suggests that feeds with long-term exposure to the antibiotic may develop altered populations which have adapted to the selective pressure, in other words, a significant sulfonamide–enriched fraction. To examine whether this might have an impact on the recovery of an incidental strain, such as OLC-2685 cells added to a sample, we studied its enrichment in three different commercially medicated feeds for which sulfonamide levels were determined using a standard analytical chemistry technique. Enrichment of the MDR model strain under fast growth conditions in the feed sample with the low level of sulfamethazine resulted in a high level (100 %) of recovery of the strain on plating media, as evidenced by the presence of the strain-specific, *Sul1* and *blaKPC-3* markers in all of the colonies assayed (*p* < 0.0001) (Table 5). However, its recovery in the feeds containing high levels of the antibiotic was significantly lower, with a recovery level of 7 % in one sample (*p* = 0.236) and no recovery in the other. When the samples were subjected to slow growth conditions (room temperature for 7 days), only the feed with a low level of sulfamethazine produced significant recovery of the inoculated model strain (*p* < 0.05) (Table 6), albeit at a lower rate (18 %) than observed in the other experiments.

### Enrichment in a Commercially Medicated Feed Under Nutritionally Limited, Moist Conditions

The previous experiments were conducted under conditions where bacteria were enriched in a nutrient-rich broth, which may not be representative of “real world” conditions where feeds and their endogenous bacteria exist in a low-nutrient environment which is stressful to bacterial cells and possibly not conducive to vigorous growth necessary for selection and enrichment to occur. Strain OLC-2685 was inoculated into a small sample of commercially medicated feed (OTT-FE-2016-0651, which had been shown to enable enrichment of the MDR model strain) moistened by the addition of sterile saline solution, and then incubated in a high moisture incubator at 37°C for 6 days. Significant background microbial growth was evidenced by the attainment of moderately high cell densities in both the uninoculated and inoculated samples, with all colonies recovered on plating media from the inoculated sample having the strain-specific, *Sul1* and *blaKPC-3* markers, and none of the colonies recovered from the uninoculated sample having these markers (*p* < 0.0001) (Table 7). These results demonstrate that selection of sulfonamide-resistant bacteria can occur under simulated feed storage conditions of high temperature and humidity that are propitious for microbial growth even in the absence of nutrient-rich media.

This study focused on obtaining preliminary evidence of co-selection of medically relevant antimicrobial resistances occurring in an MDR bacterial strain in medicated feeds. The inadvertent selection of resistance to medically important antibiotics such as carbapenem, even in common bacteria such as *K. pneumoniae*, may pose a significant public health concern since their presence in the food production chain could ultimately lead to exposure of the human microbiome to AMR bacteria which can transfer their resistance traits to pathogenic bacteria. We have effectively demonstrated that selection and enrichment of AMR bacteria can occur in feeds, and most significantly, even when feeds are medicated with antibiotics not used in human medicine co-selection of resistance to clinically significant antibiotics can occur for bacterial strains harboring multiple resistance traits.

Animal feeds are immensely variable in terms of their ingredient composition, background bacteria profiles, storage and handling conditions, and any or all of these factors may impact the extent to which selection and enrichment of AMR bacteria might occur. However, we observed significant recovery of the MDR model strain under different conditions even with a small sampling of randomly acquired feeds. Furthermore, the MDR *K. pneumoniae* model strain used in these studies is a raw sewage isolate and thus a representative denizen of the environmental microbial communities found in urban and agricultural regions of North America, and therefore highly likely to come into eventual contact with food production systems. These factors point to a high likelihood that enrichment of AMR bacteria and co-selection of clinically significant AMR traits are significant events which may contribute to the emergence of AMR bacteria to which humans are exposed through the food chain.

## Author Contributions

AC isolated the strains used in the study as well as helped with experiment planning. EB performed the recovery and bacterial screening experiments. CC helped with project planning. BB did project planning and wrote the manuscript.

## Conflict of Interest Statement

The authors declare that the research was conducted in the absence of any commercial or financial relationships that could be construed as a potential conflict of interest.

## Footnotes

1 https://github.com/OLC-Bioinformatics/COWBAT/tree/v0.1.5

2 https://cge.cbs.dtu.dk/services/ResFinder-2.1/, last [accessed 16 August, 2018]
